# Rapid cycling bipolar disorder: Literature review on pharmacological treatment illustrated by a case report on ketamine

**DOI:** 10.1002/brb3.2483

**Published:** 2022-01-18

**Authors:** Alexis Bourla, Florian Ferreri, Thomas Baudry, Vincent Panizzi, Vladimir Adrien, Stéphane Mouchabac

**Affiliations:** ^1^ Sorbonne Université, AP‐HP Department of Psychiatry Hôpital Saint‐Antoine Paris France; ^2^ Sorbonne Université ICRIN Psychiatry (Infrastructure of Clinical Research In Neurosciences ‐ Psychiatry) Brain Institute (ICM) INSERM CNRS Paris France; ^3^ INICEA Jeanne d'Arc Hospital Korian Saint‐Mandé France

**Keywords:** ketamine, pharmacological treatment, rapid cycling bipolar disorder, review

## Abstract

**Introduction:**

Rapid cycling bipolar disorder (RCBD) is defined as four or more affective episodes (depression, mania or hypomania) within 1 year. RCBD has a high point of prevalence (from 10% to 20% among clinical bipolar samples) and is associated with greater severity, longer illness duration, worse global functioning and higher suicidal risk, but there is no consensus on treatment option. The use of several pharmacological agents has been reported (levothyroxine, antipsychotics, antidepressants and mood stabilizers).

**Objective:**

The main objective of this review was to propose a critical review of the literature and to rank the pharmacological agent using a level of evidence (LEO) adapted from the Center for Evidence‐Based Medicine, and to illustrate it with a case report on off‐label intravenous ketamine.

**Method:**

We conducted a review using the MeSH terms and keywords (bipolar [Title/Abstract]) AND (rapid [Title/Abstract]) AND (cycling [Title/Abstract]) AND (treatment [Title/Abstract]). Alexis Bourla and Stéphane Mouchabac screened 638 documents identified through literature search in Medline (PubMed) or by bibliographic references and 164 abstracts were then analyzed. Nonpharmacological treatments were excluded.

**Result:**

Seventy articles were included in the review and divided into six categories: mood stabilizers, antipsychotics, hormonal treatments, ketamine and other pharmacological treatments.

**Discussion:**

Our review highlights the heterogeneity of the pharmacological treatment of RCBD and no clear consensus can emerge.

## Graphical abstract

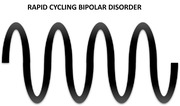

## 1 INTRODUCTION

Rapid cycling bipolar disorder (RCBD) is defined as four or more affective episodes (depression, mania or hypomania) within 1 year (Carvalho et al., 2014). It is characterized by poor response to lithium (Grunze et al., 2002) and has been reported to be a predictor of nonresponse to other usual bipolar treatments (Cruz et al., 2008). There is a clear paradox since RCBD has a high point of prevalence (from 10% to 20% among clinical bipolar samples) and is associated with greater severity, longer illness duration, worse global functioning and higher suicidal risk (Coryell et al., 2003; Kupka et al., 2005; Schneck et al., 2004), but there is no consensus on treatment option. There are few studies that have tested specific treatments for RCBD and fewer randomized controlled trials (RCT).

In a previous review, Fountoulakis et al. (2016) reported the use of several pharmacological agents: levothyroxine, antipsychotics, antidepressants and mood stabilizers. The authors conclude that there is a need for more rigorously designed studies and for newer agents. Although several evidence support effectiveness of ketamine in treatment‐resistant cases of bipolar depression (Bobo et al., 2016; McIntyre et al., 2021), there are few data on the effectiveness and risk of ketamine for RCBD.

The aim of the current review was to review published evidence assessing the efficacy of various pharmacological treatments in RCBD after a case reporting successful treatment with ketamine.

There is only one other case reporting the use of ketamine for the treatment of suicidal depression in RCBD (Sampath et al., 2016). To our knowledge, our case is the first report of maintenance treatment using multiple ketamine intravenous (IV) for RCBD. We discuss several factors that might have contributed to our patient disease and to ketamine‐induced remission since the patient was extensively investigated.

### 1.1 Case report

A 48‐year‐old Caucasian female with BD II disorder was hospitalized in our inpatient psychiatric department due to RCBD with ultradian cycling (ultra‐rapid cycling bipolar disorder [URCBD]). Earlier, she was hospitalized in a private psychiatric hospital for 6 months, and the disease was active for over 1 year.

#### 1.1.1 Previous hospitalization

One year before she was hospitalized in our psychiatric department, she was admitted to a private psychiatric hospital where multiple treatment lines were used (Table 1).

**TABLE 1:** Treatment used

Aripiprazole	Interrupted for akathisia
Quetiapine	Interrupted for sedation
Valproate	Ineffective
Lithium	Partially effective
Lamotrigine	Partially effective
Pramipexole	Effective on her restless leg syndrome
Venlafaxine	Probable hypomanic inducer at dose >75 mg. Partially effective on her depressive symptoms at 37.5 mg
Celecoxib	Possibly effective

During this period, she presented symptoms of URCBD with severe depressive symptoms in the morning and hypomanic symptoms in the early evening for several months. An association of lithium extended release (XR) 600 mg, lamotrigine 400 mg at bedtime, pramipexol 0.18 mg in the morning and venlafaxine 37.5 mg in the morning was able to partially stabilize her in a slightly dysthymic state, but she relapsed with a hypomanic episode (10 days) before a severe depression.

She was also assessed by the organo‐psychiatric team (Ferreri et al., 2019), and we find several comorbidities that might have played a role in her treatment‐resistant disease (Table 2).

**TABLE 2:** Comorbidities

Restless leg syndrome	Successfully treated with pramipexole
Severe sleep apnea	Treated with Continuous Positive Airway Pressure device (CPAP) She was not overweight and had no snoring or other typical features commonly associated with sleep apnea
Inflammatory markers	She had high levels of interleukin 2, 6 and TNF‐α
Ultra‐rapid metabolizer	She had a duplication of CYP2D6
Scintigraphic abnormalities	Severe hypometabolism in the frontal and in the amygdala‐hippocampus region of the brain on a cerebral scintigraphy
B3 vitamin deficiency	She had B3 vitamin deficiency corrected by B3 supplementation
Other	Other somatic causes were excluded after physical and neurological examination Blood morphology, electrolytes, kidney and liver profile, TSH, FT4, FT3, CRP, FAN, anti‐DNA, anti‐ENA, anti‐NMDA, B12, folate, B1, B6 levels, urine test and toxicology turned out normal

#### 1.1.2 Current hospitalization

On admission, the patient presented severe depressive symptoms with decreased mood and energy, suicidal thoughts and sleeping difficulties.

Lithium serum level was 0.6 mmol/L on dose of 600 mg/day, and lamotrigine serum levels was 6 μg/ml (4–10). The cerebral magnetic resonance imaging (MRI) showed only a small hippocampal atrophy (Schelten I).

A dose of 0.5 mg/kg ketamine hydrochloride IV infusion over a period of 40 min was given two times per week for a period of 4 weeks (*n* = 8) as add‐on treatment to her standard care (ketamine was used because esketamine was not yet accepted in France at the time of our protocol). After the first two weeks, a clear mood improvement was observed, and no hypomanic symptoms appeared. During the first doses of ketamine administration, she presented mild dissociative symptoms which resolved quickly. No other significant adverse events were observed.

After 4 weeks, she was euthymic and discharged. The IV frequency was adapted for an outpatient program (1 per week for 4 weeks, then 1 every 2 weeks for 2 months). In total, she had 16 IV in 4 months, and no adverse effect was observed. She was regularly assessed with blood tests (blood morphology, electrolytes, kidney and liver profile and TSH), which turned out normal.

At 6 months, the euthymic state still sustained, and she was able to return to the work she had left 2 years ago. A new cerebral scintigraphy was performed: the hypometabolism was no longer found. Similarly, we assessed her interleukin levels (IL‐2, IL‐6, TNF‐α) again: all three were found to be normal. At 1 year, she experienced a relapse in a moderate depressive state.

## 2 METHOD

**FIGURE 1:**
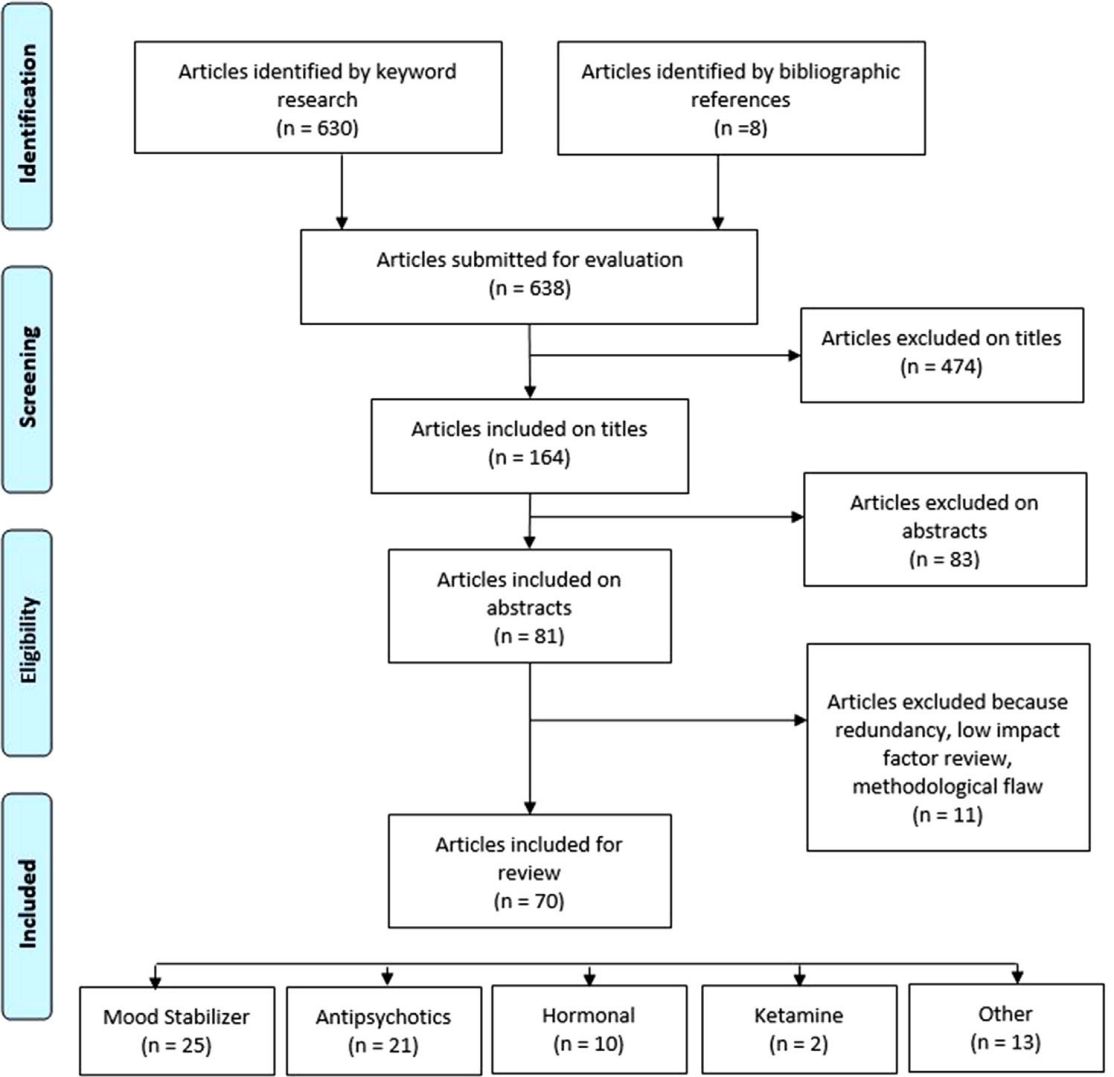
PRISMA diagram

We conducted a review using the MeSH terms and keywords: (bipolar [Title/Abstract]) AND (rapid [Title/Abstract]) AND (cycling [Title/Abstract]) AND (treatment [Title/Abstract]) until July 2021. Alexis Bourla and Stéphane Mouchabac screened 638 documents identified through literature search in Medline (PubMed) or by bibliographic references, and 164 abstracts were then analyzed. Seventy articles were included in the review (see PRISMA diagram, Figure 1) and divided into the five following categories: (1) mood stabilizers, (2) antipsychotics, (3) hormonal treatments, (4) ketamine and (5) other pharmacological treatments. Nonpharmacological treatments were excluded.

The results were ranked using a level of evidence (LEO) adapted from the Center for Evidence‐Based Medicine (Howick et al., n.d.) (see Table 3).

**TABLE 3:** Level of evidence

Definition	Quality of evidence	Code
Several high‐quality studies with consistent results At least one large, high‐quality multicenter trial	High	A
One high‐quality study Several studies with some limitations	Moderate	B
One or more studies with severe limitations Case reports	Low	C
Expert opinion One or more studies with very severe limitations or negative results	Very low	D

## 3 RESULTS

### 3.1 Mood stabilizer

#### 3.1.1 Valproate

Several case reports suggested the efficacy of valproate for RCBD (Steingard, 1989) particularly when co‐occurring with bulimia (Herridge & Pope, 1985) or refractory to treatment with lithium salts, neuroleptics or carbamazepine (McElroy et al., 1988).

Calabrese and Delucchi (1990) first reported the efficacy of valproate in a prospective open trial on 55 patients under monotherapy (*n* = 20) or combination therapy (*n* = 35). They showed moderate acute antidepressant responses in 47% of the patients and acute antimanic responses in 91%. valproate seems effective as a prophylactic therapy with prophylactic antidepressant responses in 76% and prophylactic antimanic responses in 94% of the patients. It seems mainly effective for mixed states (acute responses in 85%, and prophylactic responses in 93%). The same authors tried to replicate this study on 78 RCBD patients (Calabrese et al., 1992) 2 years later with a longer trial duration (15.8 months) and found less effective efficacy with low acute antidepressant responses (19% of the patients), moderate acute antimanic responses (54%) but high mixed state responses (87%). Similarly, the prophylactic antidepressant responses were seen in only 33% of the depressed patients and were more important for manic (72%) or mixed (94%) prevention. Based on these results, Calabrese et al. (1993) stated that valproate has marked antimanic efficacy, poor‐to‐moderate antidepressant properties and good antimixed state responses (but then became depressed).

Valproate was tested against lithium in a large RCT (20 months, double‐blind, parallel‐group) on 254 patients (Calabrese et al., 2005) but with a high discontinuation rate (76%) due to poor adherence, nonresponse or side effects. Of the 60 remaining patients, the rates of relapse into any mood episode were 56% for those given lithium versus 50% for those on valproate.

#### 3.1.2 Carbamazepine

Low evidence was found about the efficacy of carbamazepine for RCBD despite the fact that it is regularly cited as an effective treatment. Only one open trial on 12 patients was found (Joyce, 1988), and only two had a complete remission on carbamazepine alone, two had a complete remission to a combination of lithium and carbamazepine, three had low beneficial effect and five patients were unresponsive.

#### 3.1.3 Lamotrigine

Several case reports exhibit the acute antidepressant effect of lamotrigine for RCBD (Calabrese et al., 1996; Kusumakar & Yatham, 1997) in treatment‐resistant patients (to lithium, carbamazepine and antidepressant medications) at a dose ranging from 75 to 200 mg/day or in treatment‐resistant menstrually related RCBD in women (in combination with valproate and levothyroxine) (Becker et al., 2004). Two other case reports showed a positive effect of adjunctive lamotrigine to valproate in treatment‐resistant RCBD patient after 5 weeks at a dose ranging from 75 to 100 mg/day (da Rocha et al., 2007; Woo et al., 2007).

In an open, naturalistic trial on five patients, the effect persisted for an average of 7.5 months (Fatemi et al., 1997). In a double‐blind, placebo‐controlled, prophylaxis study of lamotrigine on 324 patients with RCBD, 41% of lamotrigine patients versus 26% of placebo patients were stable without relapse for 6 months of monotherapy (Calabrese et al., 2000). A prospective study assessing weekly mood shifts using a Life Chart Method (self‐report) was examined over 26 weeks in 182 RCBD patients randomly assigned to lamotrigine or placebo (Goldberg, Bowden, et al., 2008). Subjects taking lamotrigine were 1.8 times more likely than those taking placebo to achieve euthymia at least once per week over 6 months and had an increase of 0.69 more days per week euthymic compared to those taking placebo, which was statistically significant (*p* = .014) but appeared clinically irrelevant.

Lamotrigine showed little clinical efficacy on an RCT on 36 RCBD patients with comorbid substance use disorder (SUD), unresponsive to a combination of lithium and valproate, randomized to receive lamotrigine (*n* = 18) or placebo (n ± 18) (Wang et al., 2010). The changes were not statistically significant (*p* = 0.27) for depressive symptoms (Montgomery–Åsberg Depression Rating Scale [MADRS]) although they found −9.1 ± 11.2 in the lamotrigine group versus −4.5 ± 13.1 in the placebo group‐treated patients. There were no significant differences either in changes in Young Mania Rating Scale (YMRS) total scores and rates of response or remission. The same team produced a second study (Kemp et al., 2012) and concluded that lamotrigine addition to patients nonresponsive to a lithium plus valproate combination was ineffective for the majority of patients with RCBD and that lamotrigine was no more effective than placebo in reducing depression severity.

#### 3.1.4 Topiramate

One case report in a patient with a treatment‐resistant RCBD with ultradian cycling responded to adjunctive topiramate 100 mg (in combination with lithium, valproate and loxapine), and the response was maintained during a 3‐year follow‐up (Karama & Lal, 2006).

Kusumakar et al. (1999) completed an open‐label study of adjunctive topiramate in 27 female RCBD patients, refractory to two or more previous mood stabilizers for at least 12 months. Note that 55.5% of patients exhibited a clinically significant improvement in mood and achieved euthymia at week 16.

#### 3.1.5 Lithium

A 6‐month, double‐blind, parallel‐group comparison was carried out in 149 RCBD patients with co‐occurring alcohol, cannabis or cocaine abuse or dependence but almost 80% discontinued the study prematurely (Kemp et al., 2009). The rate of relapse into a mood episode for those receiving lithium monotherapy or the combination of lithium and divalproex was 56% (*N* = 9) and 53% (*N* = 8), respectively, meaning that the addition of valproate to lithium conferred no additional prophylactic benefit over lithium alone, and that lithium was not able to prevent relapse for the majority of patients.

Amsterdam et al. (2013) showed in an RCT on 37 participants with RCBD receiving initial fluoxetine randomly assigned to long‐term monotherapy with either fluoxetine 10−40 mg daily, lithium 300−1200 mg daily (with a serum level of 0.5−1.5 mmol/L) or placebo for 50 weeks. There was no significant difference between the three groups in the long‐term follow‐up, suggesting that maintenance lithium or fluoxetine monotherapy is similar to placebo during long‐term relapse‐prevention therapy for RCBD. When used in combination, lithium showed a better efficacy than used in monotherapy, particularly when associated with carbamazepine in several case reports (Di Costanzo & Schifano, 1991; Laird & Knox, 1987), or associated with valproate with a significant improvement in their depression within 24–48 h of the addition of lithium to valproate (Sharma et al., 1993).

### 3.2 Antipsychotics

#### 3.2.1 Clozapine

Efficacy of clozapine for psychotic and nonpsychotic RCBD was suggested by multiple case reports (Calabrese et al., 1991; Frye et al., 1996; Lançon & Llorca, 1996) in combination with lithium or levothyroxine (Suppes et al., 1994) with a dose ranging from 150 to 400 mg in acute phase and from 150 to 275 mg/day for relapse prevention. Another case report showed a good response to a combination of topiramate with clozapine (C. K. Chen et al., 2005), but it seems very clear that it was the addition of clozapine which has achieved remission since topiramate was previously used with several other medications (olanzapine, risperidone and quetiapine) and was ineffective. A case report also showed a good and sustainable remission (5‐year follow‐up) with a combination of lamotrigine 100 mg and clozapine 150 mg (Bastiampillai et al., 2010), and it seems clear that it was the addition of clozapine to a previously poor effective lamotrigine that induced the remission, and the authors suggest that clozapine monotherapy might have been similarly effective.

A retrospective study on 13 RCBD patients, with a dose ranging from 25 to 600 mg/day (mean 180 mg/day), showed statistically significant changes in the number of days with depression, days with mania, depressive episodes, manic episodes, hospitalizations and suicide attempts (Kılınçel et al., 2019).

In a prospective randomized (clozapine add‐on vs. treatment as usual) study on patients with bipolar disorder (*n* = 20) or schizoaffective disorder (*n* = 8) experiencing rapid cycling (*n* = 15) or not (*n* = 13), Suppes et al. (2004) found that clozapine was more effective for nonrapid‐cycling affective disorder than for RCBD, although RCBD improved quickly (−14.8 points in Brief Psychiatric Rating Scale [BPRS] in the first month) but worsened by 0.1 point per month after the first month, while nonrapid‐cycling improved by 1.3 points per month: BPRS score at 32 points for RCBD after 12 months versus 23 points for nonrapid‐cycling.

#### 3.2.2 Quetiapine

A case report showed improvement in an RCBD patient with comorbid obsessive‐compulsive disorder (OCD) and lithium‐induced movement disorder (i.e., tremor) at the dose of 600 mg with a very good effect on the movement disorder which disappeared within 1 week, and a quick (2 weeks) and sustainable (6 months) response (Stratta et al., 2003). Valerius et al. (2005) reported a significant improvement at the dose of 200 mg for an RCBD woman with comorbid anxiety and social phobia which also improved, but prior treatment for that patient seems poorly adapted (fluphenazine, perazine and flupenthixol) to RCBD.

A prospective open‐label study assessed the impact of add‐on quetiapine in 14 RCBD patients and found a statistically significant reduction in the YMRS (−1.01 point) but not in HDRS or Clinical Global Impressions‐Bipolar Scale (CGI‐BP) (Vieta et al., 2002). Another open‐label, nonrandomized trial was conducted on 41 RCBD patients who received quetiapine monotherapy (*n* = 19) or add‐on therapy (*n* = 22) for 1 year (Goldberg, Kelley, et al., 2008). This study showed a greater efficacy on manic symptoms than on depressive symptoms and a larger effect size in the add‐on group, but only 32% of the patients finished the study without the need of additional psychotropic treatment.

Quetiapine was compared with valproate in an open‐label, parallel group, multicentric, 12‐months trial on 38 remitted or partly remitted RCBD patients, randomized in valproate (*n* = 16) or quetiapine (*n* = 22) monotherapy (Langosch et al., 2008). They found that quetiapine was more effective than valproate in the treatment of RCBD, specially for the number of depressive days but not for the treatment of manic symptoms. Furthermore, quetiapine was less tolerated than valproate, although more patients in the quetiapine group completed the trial.

#### 3.2.3 Olanzapine

Olanzapine showed in several RCT that it is less effective in RCBD patients than in nonrapid‐cycling (Vieta et al., 2004), but it is more effective than placebo (Baldessarini et al., 2003; Sanger et al., 2003; Shi et al., 2004) and may be as effective as valproate (Suppes et al., 2005).

#### 3.2.4 Other antipsychotics

Almost every second‐generation antipsychotic was tested in RCBD in clinical trials, including risperidone (Vieta et al., 1998), risperidone long‐acting injection (LAI) in an RCT showing that it is ineffective (Bobo et al., 2011). Aripiprazole was tested against placebo on 12 RCBD (five in the placebo group) over a 26–100 weeks period, and the study showed that aripiprazole significantly delayed the time to relapse (Muzina et al., 2008).

### 3.3 Hormonal treatment

#### 3.3.1 Levothyroxine

Stancer and Persad (1982) successfully treated RCBD patients with high dose of levothyroxine (up to 0.5 mg/day with a starting dose of 0.1 mg/day raised every week to achieve the maximum dose in 5 weeks). This treatment led to remission on five of seven women with treatment‐resistant RCBD (including lithium, antidepressant, neuroleptics, and electroconvulsive therapy [ECT]). This effect was also found in several case reports with efficacy including in a woman with ultradian cycling/URCBD (hypomanic in the morning and drowsy and depressed in the afternoon) (Leibow, 1983). A preliminary study on 11 RCBD patients (10 women and one man) found a clear response of depressive symptoms in 10 of 11 patients, and manic symptoms response in five of seven patients who exhibited symptoms during baseline evaluation (M. S. Bauer & Whybrow, 1990). When four patients underwent placebo substitution (single‐ or double‐blind), three of four relapsed into either depression or cycling. In that study, all patients were resistant to lithium, some were resistant to a lithium plus carbamazepine combination, and one patient was resistant to monoamine oxidase inhibitors (MAOI). The dose range of levothyroxine in this study was 0.15–0.4 mg/day. This study was also the first with successful use of levothyroxine on a man, although he relapsed after 6 months (but probably because he decided to decrease his lithium dose). Efficacy on another man was found in another case report with quick response (1 week) using only 25 μg of T4/day (Bernstein, 1992) and 50 μg of levothyroxine on a man having subclinical hypothyroidism (TSH 6.36 μIU/ml) (P. H. Chen & Huang, 2015). While previous study showed no correlation between thyroid status and treatment response, Baumgartner et al. (1994) found in an open clinical trial on six patients that four of six patients have a history of subclinical hypothyroidism and/or Hashimoto's thyroiditis, and that the physiological dose of T4 was insufficient for these patients, while supraphysiological doses led to remission. Supraphysiological dose of levothyroxine was also found to be effective in an open trial on six RCBD with two remissions and two partial responses (Afflelou et al., 1997).

Weeston and Constantino (1996) reported successful treatment of a severe RCBD 13‐year‐old patient who failed adequate trials of lithium, carbamazepine, valproate, a variety of neuroleptics, calcium channel blockers, nortriptyline, paroxetine, clomipramine, clonidine and ECT in which 125 μg/day of levothyroxine was well supported and led to significant remission.

Finally, the first (and only to date) comparative double‐blind, placebo‐controlled trial in 32 RCBD patients receiving either L‐T4 (*n* = 13), L‐T3 (*n* = 10) or placebo (*n* = 9) found that the L‐T4 group spent a significantly greater time in a euthymic state (+33.1%) compared to other groups (T3 and placebo groups did not differ significantly). The L‐T4 group spent significantly less time depressed (−18.1%, *p* = .022) or in a mixed state (−13.3%, *p* = .031) compared to pretreatment (Walshaw et al., 2018).

#### 3.3.2 Melatonin

In a double‐blind, placebo‐controlled trial (Leibenluft et al., 1997), five RCBD patients were treated with melatonin 10 mg at 10:00 p.m. for 12 weeks in addition to their previous medication (melatonin was added to a stable regimen). This trial report showed no positive effects, while melatonin withdrawal delayed sleep onset time and might have some mild mood‐elevating effects.

### 3.4 Ketamine

We found only one case report supporting the use of ketamine for RCBD (Sampath et al., 2016): a 19‐year‐old female with suicidal depression resistant to lithium and quetiapine who refused ECT and was treated with ketamine IV, 0.5 mg/kg in 100 ml saline over 40 min. MADRS changed from 36 to 18 twelve hours after ketamine IV. A second ketamine IV was administered after 5 days and at week 3. The MADRS score was 6, meaning that her depression remitted. She remained stable at the 4‐month follow‐up, but that study focuses essentially on ketamine for the rapid and effective relief of suicidal depressive ideation in RCBD.

### 3.5 Other pharmacological treatments

Several other pharmacological agents have been tested for RCBD (Amann et al., 2007; Bräunig & Krüger, 2003; Chouinard et al., 1990; Davanzo et al., 1999; Goodnick, 1995; Haykal & Akiskal, 1990; Hegde et al., 2015; Keck et al., 2006; Lyoo et al., 2003; Mah & Conn, 2010; Sharma & Barrett, 2001; Stoll et al., 1996; Sugimoto et al., 2003). Results of case reports or clinical trials are summarized in Table 4.

**TABLE 4:** Other pharmacological treatments

Study reference	Treatment	Method or procedure	Result
Oomen et al. (1996)	Magnesiocard	Pilot‐study on nine RCBD women 8 weeks open label Age range: 35–52 years 8‐month follow‐up Magnesiocard 40 mEq/day Added to lithium (previously ineffective)	Six of nine patients were successfully discharged within 8 weeks, but three of them could not be maintained as outpatients at 6‐month follow‐up
Pestana et al. (2018)	Bupropion	Case reports (open clinical observation) on four women and two men with RCBD type 2 Age range: 25–45 years Two‐year follow‐up Bupropion	Significant improvement in all six patients Dramatic and sustained response in four of six
Sampath et al. (2016)	Nimodipine	Case reports on one woman (53 years) and one man (58 years) with RCBD One‐year follow‐up Nimodipine 180 mg/day	Relapse prevention was achieved for 12 months (for the woman) and five months (for the man)
Sanger et al. (2003)	Nimodipine	Case report on a 13‐year‐old boy with refractory RCBD (ultradian cycling) Nimodipine 180 mg/day	Remission was observed and measured by standardized scales after 9 days of treatment and was sustained at 36‐month follow‐up Bias: adjunctive treatment with levothyroxine
Schneck et al. (2004)	Choline	Case report on six outpatients RCBD In combination with lithium Treatment: choline 2000–6000 mg/day	Significant improvement in four of six patients. Two patients who did not initially improv Choline was also the only two cases receiving supratherapeutic Thyroxin and one of these patients did appear to respond well following the deliberate discontinuation of this thyroxine
Sharma and Barrett, 2001	Choline	RCT choline versus placebo On eight lithium‐resistant RCBD patients In combination with lithium Treatment: choline 50 mg/kg 12 weeks follow‐up Brain purine level was assessed using MR‐spectroscopy	No significant differences in change‐from‐baseline measures of CGIS, YMRS and HDRS over a 12‐week assessment period between groups. However significant decrease in brain purine levels was found
Sharma et al. (1993)	Tryptophan	Case report on a 40‐year‐old woman with RCBD and comorbid fibromyalgia resistant to several antidepressant, lithium, valproate and carbamazepine used alone or in combination Treatment: L‐tryptophan gradually increased at 4 g/day in addition with lorazepam 1 mg and oxazepam 25 mg	Mixed state after 2 weeks at 4 g/day, and significant improvement (mood stability for at least 18 months) after reducing the dosage at 2 g/day. Fibromyalgia symptoms were also improved
Shi et al. (2004)	Clonazepam	Case report on a 14‐year‐old man with RCBD (10 days cycling with severe mania accompanied by auditory hallucinations) resistant to carbamazepine, valproate and lithium used alone or in combination Treatment: clonazepam increased to 11 mg/day (serum level 50 μg/L) in addition to a combination of valproate + lithium	Complete remission with 1‐year follow‐up. No adverse effects were noted
Stancer and Persad, 1982	Levetiracetam	Tow case reports: on a 49‐year‐old woman resistant to valproate and “several add‐on strategies” with a severe depressive episode Treatment: levetiracetam 500 mg and increased to 2000 mg/day was added to a combination of valproate 2500 mg/day, lorazepam 4 mg and zotepine 7.5 mg	In the first case report, depression remitted after 4 weeks and the patient became slightly euphoric for 6 weeks before achieving euthymia for 7 weeks. Afterward, she developed dysphoria before reachieving euthymia with no relapse at the 6‐month follow‐up
		On a 51‐year‐old man resistant to carbamazepine, valproate, lamotrigine and several add‐on antipsychotics (risperidone, olanzapine and quetiapine) in a mixed manic state with psychotic and catatonic features Treatment: levetiracetam 500 mg and rapidly increased to 2000 mg/day was added to a combination of valproate 1500 mg/day, lorazepam 2 mg and olanzapine 20 mg	In the second case, full remission of the mixed mania was achieved within 3 weeks and remained stable at the 5‐month follow‐up
Steingard, 1989	EPA	RCT EPA versus placebo Four‐month RCT on RCBD men and women (20–73 years old) randomized in adjunctive trial of ethyl‐eicosapentanoate (EPA) 6 g/day (*n* = 31) or placebo (*n* = 28)	No significant differences were found between the two groups
Stoll et al. (1996)	Chromium	Two years open label study on 30 RCBD patients (14 females and 16 males, mostly type I BD) resistant to at least 6 months of treatment including mood stabilizers, antipsychotics and antidepressants Treatment: 600 to 800 μg/day of hypoallergenic chromium	Thirty‐nine percent of patients were considered responder after 3 weeks on the MADRS. Seven patients could be followed up for 1 year, and six of them showed a reduction in the mean number of affective episode (from 6 to 2.6) High number of dropout while paradoxically chromium was very well tolerated
Stratta et al. (2003)	Pramipexole	Case report on a 77‐year‐old woman type I RCBD (depressive and hypomanic episode cycling for 5 years) resistant to lithium, gabapentin, valproate, several antidepressants including MAOI, several antipsychotics and ECT Treatment: 0.25 mg/day of pramipexole added to current treatment (combination of bupropion, lamotrigine, levothyroxine and estradiol)	Improvements were found after 8 weeks: no more cycling but persistent anhedonia. Pramipexole was slowly increased at 0.75 mg/day over 10 weeks and lad to further improvement but with residual symptoms
Sugimoto et al. (2003)	Pramipexole	Case report on a 37‐year‐old man with a 12‐year history of type II RCBD with catatonic features resistant to lithium, lamotrigine, fluoxetine, olanzapine and ECT Treatment: 0.125 mg/day, hiked up to 0.5 mg/day in two divided doses over 2 weeks	Significant improvement in symptoms from the second week of initiation of pramipexole. HDRS score improved from 22 to 7 by the end of 1 month of treatment. The Bush‐Francis Catatonia Rating Scale scores reduced from 8 to 0 during the same period. The improvement persisted at 2‐month follow‐up, and no adverse effects were reported

Abbreviations: CGIS, Clinical Global Impression Scale; ECT, electroconvulsive therapy; HDRS, Hamilton Depression Rating Scale; MAOI, Mono‐Amine Oxidase Inhibitor; RCT, randomized controlled trial; YMRS, Young Mania Rating Scale.

## 4 DISCUSSION

RCBD is associated with poorer clinical outcomes, and some clinical features such as early onset, number of lifetime mood episodes, and cognitive deterioration emphasize the burden of rapid cycling (Pestana et al., 2018).

Our review highlights the heterogeneity of the pharmacological treatment of RCBD, and no clear consensus can emerge. We summarized our finding in Table 5.

**TABLE 5:** Level of evidence for pharmacological rapid cycling bipolar disorder (RCBD) treatment

Grade	Medication	Discussion
A	Levothyroxine	One positive RCT, multiple positive case reports
	Clozapine	One positive RCT, one positive retrospective study, multiple positive case reports
B	Valproate	One positive open trial, multiple positive case reports One RCT showing moderate efficacy
	Quetiapine	One positive prospective open‐label One positive open‐label, parallel group, multicentric trial
	Aripiprazole	One positive RCT
	Olanzapine	One RCT showing similar efficacy than valproate
C	Ketamine Pramipexol Topiramate Aripiprazole Bupropion Nimodipine Choline Tryptophan Clonazepam Levetiracetam Chromium	Multiple positive case reports
	Carbamazepine	One open trial showing moderate efficacy
	Magnesiocard	One pilot study showing moderate efficacy in the acute phase
D	Lithium	Two negative RCT, lithium might be effective as ‘‘add‐on’’ treatment
	EPA Melatonin Risperidone	One negative RCT
	Lamotrigine	Several positive case reports as ‘‘add‐on’’ treatment One positive open naturalistic trial, One positive open prospective study One negative RCT

Abbreviations: EPA, ethyl‐eicosapentanoate; RCT, randomized controlled trials.

There are several limitations of these studies: they all had a small sample size, the majority were not RCT, several RCT lacked placebo‐control group, the majority had a short follow‐up duration.

The most promising agent seems to be levothyroxine, and this could be explained by thyroid dysfunction and antithyroid antibodies that have been reported to be associated with mood disorders, and specifically RCBD (Gan et al., 2019). Levothyroxine (T4) and Liothyronine (T3) are widely used as an add‐on treatment and proved to be effective in improving treatment response in euthyroid patients with refractory or bipolar depression (M. Bauer et al., 1998) or bipolar depression (M. Bauer et al., 2005) probably by modulating the brain serotonin system (Mason et al., 1987), which might partly explain the role of thyroid hormone in the pathophysiology of affective disorders. An association between autoimmune thyroiditis (TPO or TG‐abs) or hypothyroidism and RCBD was observed in several studies (M. S. Bauer et al., 1990; Oomen et al., 1996), and antithyroid antibodies might influence mental well‐being regardless of thyroid dysfunction (Mussig et al., 2012).

Ketamine is a mixture of equal amounts of two enantiomers (esketamine and arketamine) that act preferentially on certain systems. It is mainly an inhibitor of the N‐methyl‐d‐aspartic acid (NMDA) receptors of glutamate. This effect is observed especially in the prefrontal cortex, thalamus and hippocampus regions involved in memory and consciousness, which explains its effects on these dimensions. However, we should not reduce its functioning to this simple NMDA blockade. There is also an antagonism on delta‐ and mu‐opioid receptors and opioid potentiation. It can increase the release of aminergic neuromodulators (dopamine and noradrenaline) by inhibiting norepinephrine transporter (NET) and dopamine transporter (DAT), it is also an antagonist of nicotinic receptors to acetylcholine. It is active in neurosteroids pathway. All these systems interact together for a global effect. These immediate effects can explain what we observe clinically during a ketamine infusion, but it seems more relevant to focus on the durable effects, such as the modification of gene expression and the effects on protein regulation. For example, we can suppose that the activation of brain‐derived neurotrophic factor (BDNF) receptor TrkB could represent a pathway for a sustainable functional and structural synaptic plasticity.

BD is considered as being a multisystem disorder particularly regarding the association with autoimmune and inflammatory disorders. In 2015, compared with healthy controls, Munkholm et al. (2015) found higher levels of IL‐6 and IL‐18 and high variability in TNF‐α dosage in patients with bipolar manic/hypomanic disorder. They concluded that these findings support a role of the altered peripheral immune response in RCBD. In a large cohort, some authors found that in a subgroup of patients, a CRP gene variant is associated with thyroid disorders and mostly with rapid cycling, especially in women (Boukouaci et al., 2018). Another study found higher levels of IL‐8, MCP‐1, IFN‐γ, IL‐6, TNF‐α in nonresponder depressed bipolar patients compared to responders (Benedetti et al., 2017).

Furthermore, some recent studies emphasize the anti‐inflammatory properties of ketamine and its ability to inhibit the systemic production of pro‐inflammatory cytokines that have been proposed experimentally (HMGB‐1, IL‐6 or TNF‐α) and more recently for clinical practice. The latter modify the metabolism of tryptophan by producing quinolinic acid (glutamate agonist) and kinuyrenic acid (glutamate antagonist) while reducing its transformation into serotonin. Thus, the activity on this pathway could be an explanation of the potential effect of ketamine in RCBD (Verdonk et al., 2019), knowing that some of these biomarkers show promise in bipolar disorders (Munkholm et al., 2019).

In definitive, for now, the evidence is too sparce to recommend the use of ketamine for RCBD since there is only two positive case reports (including our own).Since RCBD is associated with one of the most severe outcomes across the bipolar spectrum, it is worth to consider for treatment‐resistant RCBD patients.

### PEER REVIEW

The peer review history for this article is available at https://publons.com/publon/10.1002/brb3.2483

## Data Availability

Data that support the findings of this review and custom code used to perform the review are available from the corresponding author upon reasonable request.
